# Is endolymphatic hydrops, as detected in MRI, a truly cochleocentric finding?

**DOI:** 10.3389/fneur.2024.1477282

**Published:** 2024-12-02

**Authors:** Marta Álvarez De Linera-Alperi, Pablo Dominguez, Melissa Blanco-Pareja, Pablo Menéndez Fernández-Miranda, Raquel Manrique-Huarte, Gloria Liaño, Nicolas Pérez-Fernández, Víctor Suárez-Vega

**Affiliations:** ^1^Department of Otorhinolaryngology, Clínica Universidad de Navarra, Madrid, Spain; ^2^Department of Radiology, Clínica Universidad de Navarra, Pamplona, Spain; ^3^Department of Radiology, Hospital Universitario Rey Juan Carlos, Madrid, Spain; ^4^Escuela Politécnica Superior, Universidad CEU, Madrid, Spain; ^5^Department of Otorhinolaryngology, Clínica Universidad de Navarra, Pamplona, Spain; ^6^Department of Radiology, Clínica Universidad de Navarra, Madrid, Spain

**Keywords:** Meniere disease, endolymphatic hydrops, endolymphatic and perilymphatic space, 3D-real-IR MRI, vestibular pathology

## Abstract

**Introduction:**

The most common histopathological finding in Ménière’s disease (MD) is endolymphatic hydrops (EH), which involves the dilation of the membranous labyrinth. The direct relationship between EH and MD is debated, although EH plays a crucial role in auditory and vestibular functional tests. MRI sequences such as 3D-FLAIR and 3D-real-IR are used to study EH, with the latter being more effective. This study aimed to examine whether the severity of EH detected by MRI is always more pronounced in the cochlea than in the vestibule, indicating a cochleocentric progression of the condition.

**Methods:**

A retrospective longitudinal study was conducted at a tertiary care medical center from 2019 to 2023, involving patients diagnosed with unilateral Ménière’s disease. All patients underwent MRI hydrops assessments (3D-REAL-IR sequences) using 3 Tesla magnets and gadobutrol contrast agent. EH was graded qualitatively and quantitatively for both ears using scales for cochlear endolymphatic hydrops (cEH) and vestibular endolymphatic hydrops (vEH). Volumetric measurements of the vestibule and endolymph were performed, and the vestibular endolymphatic ratio (vELR) was calculated. The degree of perilymphatic enhancement (PE) and endolymphatic herniation was also assessed. Patient data, including demographics, disease features, comorbidities, hearing loss, and vestibular function, were collected from medical records. Statistical analysis involved various tests to compare groups and evaluate correlations, using a significance level of *p* < 0.05. The study aimed to classify the patients into cochleocentric (CC) or non-cochleocentric (NCC) groups based on the difference in the severity of EH in both compartments.

**Results:**

We included 137 patients, of whom 55 (40.15%) were classified as CC, and the remaining 82 (59.85%) were classified as NCC. The degree of vestibular EH (vEH) was more severe in the NCC group (*p* < 0.001), while cochlear EH (cEH) showed a moderate correlation with vEH. The mean vestibular endolymphatic ratio (vELR) was higher in the NCC group (80.5% ± 38%) compared to the CC group (55% ± 49.5%) (*p* < 0.0001). Vestibular herniation was more common in the NCC group, while vestibular perilymphatic enhancement was more prevalent in the CC group. Cardiovascular risk was associated with the CC group, while the NCC group reported more vestibular symptoms. Delayed Ménière’s disease was linked to the CC group. The hearing loss and vestibular function tests did not show significant differences between the groups.

**Discussion:**

In conclusion, our study found that endolymphatic hydrops (EH) was more severe in the vestibule than in the cochlea in nearly 60% of the cases, with a clinical correlation to the initial symptoms. However, no significant differences were observed in the auditory or vestibular function tests during the follow-up.V Previous studies have indicated that vestibular EH occurs early in Ménière’s disease (MD) and subsequently progresses to the cochlea, a finding that challenges the traditional cochleocentric progression theory supported by experimental and clinical otopathology. MRI techniques have enhanced the detection of EH, revealing that the relative amount of endolymph is slightly higher in the vestibule than in the cochlea, thereby supporting the study’s findings. We considered the important technical limitations in the MRI visualization of EH and suggested that advanced imaging techniques and volumetric quantification could enhance the classification of cochleocentric and non-cochleocentric groups. The clinical findings revealed that cardiovascular risk factors and delayed MD phenotypes were more common in the cochleocentric group, while the non-cochleocentric group exhibited poorer vestibular MRI results and a higher incidence of endolymph herniation into the semicircular canals.

## Introduction

Ménière’s disease (MD) is a chronic and progressive disorder that affects the inner ear, developing over time and worsening gradually (1). It is characterized by episodes of vertigo, accompanied by fluctuating low-frequency hearing loss, tinnitus, and aural pressure at the onset, coinciding with critical episodes (2). As the disease progresses, it leads to a functional deficit, initially affecting auditory function and later vestibular function, which can become irreversible. Additionally, it causes a significant deterioration in the patient’s quality of life due to recurrent vertigo attacks, which eventually may decrease but often result in instability that can have very diverse degrees of functional impact on the patient (3).

The complexity of the etiology is largely due to its strong association with other conditions, some of which, rather than being mere comorbidity factors, can act as true triggers and exhibit a bidirectional influence, such as migraine, autoimmune diseases, asthma, hypovitaminosis D, and osteoporosis. In the familial form, the most common alteration occurs in genes that mark the ultrastructure of the inner ear (4). In the sporadic form, anomalies have been detected in genes that are also altered in patients with sensorineural hearing loss, as well as in genes that encode proteins involved in directed axonal growth. These patients also show various epigenetic alterations involved in the regulation of inflammatory and immune responses, resulting in increased susceptibility to aggressive inflammatory processes and, ultimately, endolymphatic hydrops (EH) due to stress. This could also explain two characteristics of the disease: its progressive nature and fluctuations (5). To date, no markers have been identified, but this is the area of research where two populations have been differentiated based on the levels of IL-1β and TNF-*α* (6).

The most common histopathological finding in the inner ear of patients with MD is endolymphatic hydrops (EH), which is the dilation or distension of the membranous labyrinth due to an increase in the volume of endolymph compared to perilymph. Unlike the high variability of clinical manifestations, histological alterations in experimental animals are more organized and possibly cochleocentric: EH starts at the cochlear apex and progresses to the maculae and semicircular canals. In humans, this is a debated issue because the histological lesion resulting from secondary damage to hydrops (rupture of labyrinthine membranes) is common and appear to follow a distinct cochleocentric pattern in the cochlea, where there is a progression from the apex to the base (7). However, in the vestibular system and according to the most recent microCT studies, this does not seem to be the case. This discrepancy is due to the presence of two key structures: (1) Hensen’s duct (ductus reuniens), which connects the cochlea and saccule and could be obstructed by otoconial remnants from the saccule (8), and (2) the utriculo-endolymphatic valve (of Bast), which could control the “reflux” of endolymph toward the utricle (9). The endolymphatic sac also shows anomalies as it is frequently hypoplastic.

From a pathophysiological point of view, it is difficult to confirm the direct relationship between endolymphatic hydrops and MD, which raises questions about whether it is merely an epiphenomenon with significant effects on auditory and vestibular function tests or if it is a necessary “*sine qua non*” element upon which other factors must act (10).

Currently, two specific MRI sequences have been established for the study of EH, known as MRI hydrops: the 3D-FLAIR sequence (fluid-attenuated inversion recovery) and the 3D-real-IR sequence (real inversion recovery reconstruction). Regarding gadolinium (Gd) contrast, there are two methods of administration—intravenous and intratympanic—that show improved effectiveness in detecting EH (11). However, there is a temporal limitation because the optimal time to obtain the MRI study is 4 h after a single dose of intravenous Gd and 24 h after intratympanic instillation (12). Although the 3D-FLAIR sequence is the most commonly used, several studies have highlighted the superiority of 3D-real-IR in evaluating EH, especially cochlear EH (cEH), as it allows for better differentiation between the endolymph (which appears as a black signal) and the surrounding bone (which appears as an intermediate gray signal) in the images (13).

To assess the severity of EH, visual qualitative scales are most commonly used. Images are evaluated in a single slice, specifically in the plane that passes through the middle third of the vestibule and includes the horizontal semicircular canal. The most widely used scales are the 3-grade scale for cochlear EH (cEH) (which classifies severity as none, mild, and severe) and the 4-grade scale for vestibular EH (vEH) (which categorizes severity as none, mild, moderate, and severe).

In conclusion, while magnetic resonance imaging of hydrops is only part of the diagnostic criteria for the disease in some cases (14), it serves as a fundamental tool that, when combined with the rest of the audio-vestibular tests, can help the otoneurology specialist in evaluating the severity of the disease and determining an appropriate treatment plan.

In this study, we aimed to analyze whether the severity of EH, as detected through MRI, is always more pronounced in the cochlea than in the vestibule, indicating a cochleocentric progression.

## Materials and methods

### Patients

A retrospective longitudinal study was carried out at a tertiary care medical center between 2019 and 2023, including patients who met the established criteria for the diagnosis of definite unilateral Ménière’s disease (2). All patients underwent MRI assessments for hydrops. The following variables were analyzed: past medical history, clinical features, hearing loss, and vestibular function.

The Research Ethics Committee of the University of Navarra (project number 2021.199) approved this study. All patients included in this study provided explicit consent for the use of their data for research purposes, and written informed consent was obtained from all participants.

### MRI

**Figure 1:**
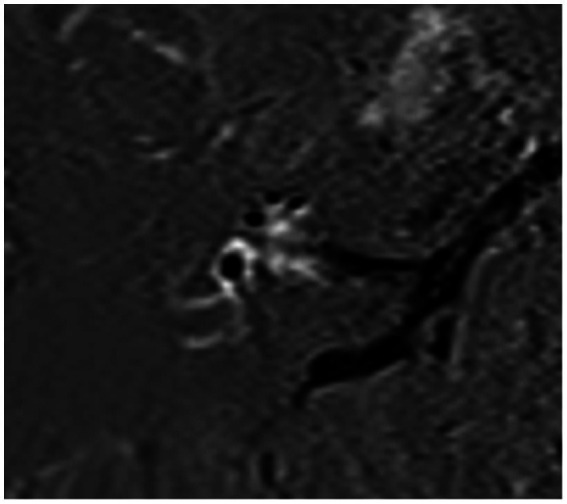
Endolymphatic MRI of a patient with unilateral MD, showing more severe hydrops in the cochlea than in the vestibule.

All MR assessments were performed using 3 Tesla magnets, either a Magnetom Vida or a Magnetom Skyra (Siemens Healthineers, Erlangen, Germany), with 20-channel and 32-channel phased-array receiver coils, respectively. The patients lay in a supine position. The images were acquired 4 h after the administration of a single dose of intravenous paramagnetic contrast agent gadobutrol (0.1 mmoL/mL, Gadovist, Bayer AG, Zurich, Switzerland) at a dose of 0.1 mL per kg of body weight (Figure 1).

#### Hydrops sequences

The hydrops imaging protocol consisted of a heavily T2-weighted sequence or “cisternography sequence” T2 3D sampling perfection with application-optimized contrast using different flip-angle evolution (SPACE) and the 3D-REAL-IR sequence. A detailed description of the sequence parameters can be found in Table 1.

**Table 1:** Main characteristics of the different MR sequences used in this study.

Sequence	T2 SPACE	3D IR
Slice thickness (mm)	0.5	0.8
Slices	56	112
Field of view (mm)	160×160	134×200
Resolution (pixels)	320×320	259×384
Voxel size (mm)	0.5 × 0.5 × 0.5	0.5 × 0.5 × 0.8
TR (ms)	1,400	16,000
TE (ms)	152	551
TI (ms)	N/A	2,700
Flip angle	120	140
Bandwidth (Hz/Px)	289	434
Acquisition time (min:s)	4:44	10:56

#### Qualitative and quantitative hydrops grading

For the assessment of the degree of EH, both ears were evaluated, and the degrees of vEH and cEH were separately recorded for each of the inner ears.

The degree of cEH was determined using a three-level scale ranging from grades 0 to 2 (none, mild, and severe), with the anatomical reference plane being the axial section passing through the modiolus, as previously reported (15). For the assessment of vEH, a four-level scale was used, with grades ranging from 0 to 3 (none, mild, moderate, and severe) and the optimal visualization plane encompassing the greatest anatomical extent of the vestibule, often aligning with the plane of the horizontal semicircular canal (16–18). The volumetric measurement of the vestibule was performed in a semi-automated manner using the cisternography sequence (T2 SPACE), while the volume of vEH was measured using the 3D REAL-IR sequence. For each patient, four different volumes were calculated: the volumes of the right and left vestibule (vT) using the cisternography sequence (two measurements) and the volumes of right and left endolymph (vE) using the 3D-REAL-IR sequence (two measurements). All volumetric measurements were performed using the advanced visualization software Siemens Syngo.via version VB50B (Siemens Healthineers), as previously reported (19). Once the volumes were obtained, the vestibular endolymphatic ratio $\left(\mathrm{vELR}\right)$ was calculated as a percentage using the following formula:


$$\mathrm{vELR}=\left(vE/vT\right)\times 100$$

**Figure 2:**
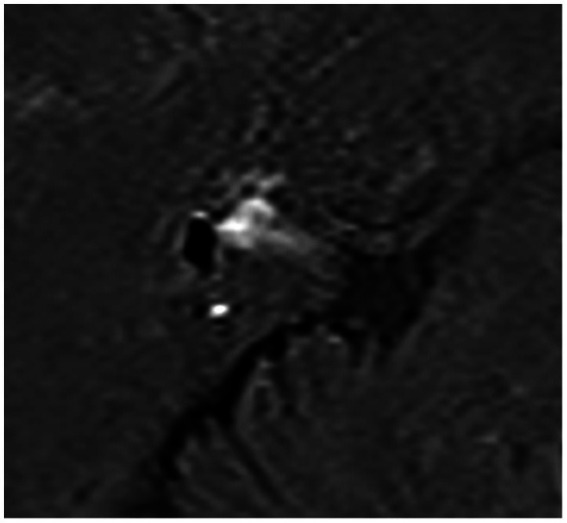
Endolymphatic MRI of a patient with unilateral MD, showing more severe hydrops in the vestibule than in the cochlea.

The degree of perilymphatic enhancement (PE) was also recorded semi-quantitatively in all ears. Radiological window settings for the 3D-REAL-IR sequence were Center 38 and Width 177-pixel intensity. Marked hyperintensity of perilymph was recorded as positive when compared to the contralateral ear, separately for the vestibule and the basal turn of the cochlea (20). The evaluation of vestibular PE was considered non-applicable in severe grade 3 vEH, as there was no perilymph left to evaluate (Figure 2).

In the 3D-REAL-Ir sequence, when there was clear evidence of herniation of the endolymph into the normally bright signal of the semicircular canal, it was classified as positive for the presence of endolymphatic herniation (21).

In this study, we assumed that EH was cochleocentric (CC group) when the severity of cEH was similar to or one degree above that of vEH. The non-cochleocentric group (NCC group) consisted of patients in whom vEH was one degree above that of cEH. In cases where both cEH and vEH were severe, the CC group was considered if there was vestibular herniation.

### Past medical history and clinical features

Patient information was obtained by reviewing the complete medical history of each patient. All clinical histories were recorded during each medical visit through the clinical follow-up for the patients with Ménière’s disease. The information collected included the following aspects:

- Demographic information: sex, date of birth, and age at the onset of disease.
- Features related to inner ear disease: disease duration, number of vertigo spells in the 6 months prior to evaluation (instability or vertigo spells lasting less than 20 min were not considered), symptom onset (synchronous, auditory symptoms, or vestibular symptoms), reason for consultation (unclear, falls, vertigo, fluctuating hearing loss, unsteadiness, benign paroxysmal positional vertigo with poor evolution, or progressive hearing loss), presence or absence of Tumarkin’s otolithic crisis, and the Ménière disease subgroup (not defined, classic, delayed, familiar, with migraine, and autoimmune).
- Features related to other comorbidities: headache (absent, migraine, or tension-type), arterial hypertension, diabetes mellitus, hypercholesterolemia, and cardiovascular risk factors.

### Hearing loss and vestibular function

All patients underwent clinical, audiological, and otoneurologic examinations.

The hearing tests included pure tone audiometry (PTA), determined by the average of four frequencies (0.5, 1, 2, and 3 kHz), as well as low-frequency (0.25–0.5 kHz) and high-frequency (4–8 KHz) PTA. The presence of spontaneous nystagmus was recorded using videonystagmography. The cervical and ocular vestibular evoked myogenic potentials (VEMP) asymmetry and the video head impulse test (vHIT) were performed on all patients.

### Statistical analysis

Quantitative variables were described using the mean and standard deviation or the median and interquartile range, as appropriate. Qualitative variables were described using the sample size and percentage.

Hypothesis contrasts for the quantitative data that were not normally distributed with similar shapes and spreads were performed using a Mann–Whitney U test, while Mood’s Median test was used when the distributions had different shapes and spreads. Statistical differences in categorical variables were assessed using the chi-square test with Monte Carlo simulation. In the case of a positive test when comparing more than two groups, *post hoc* contrasts were performed using the same statistical tests. Finally, correlation analysis between the ordinal variables vEH and cEH was conducted using Kendall’s Tau. A *p*-value of <0.05 was considered statistically significant. The ability vELR to classify patients was evaluated using a receiver operating characteristic (ROC) curve. This analysis and the Youden Index were also used to estimate the optimal cut-off point. The sensitivity and specificity of vELR, using the optimal cut-off point, were estimated to assess its potential for clinical use.

Statistical analyses were conducted using R version 4.1.0.

## Results

This study enrolled 137 patients, including 71 (51.82%) male and 66 (48.18%) female patients, with a median age of 56 ± 17 years.

The patients were classified into two groups according to the degree of endolymphatic hydrops. A total of 55 (40.15%) patients were included in the CC group, while the remaining 82 (59.85%) were included in the NCC group.

In Tables 2, 3, we present a summary of the findings and the corresponding statistical comparisons.

**Table 2:** Results of the chi-square tests for the qualitative variables.

Variable	Total	Groups		*p*-value
		Cochleocentric	Non-cochleocentric	
	*n* (%)	*n* (%)	*n* (%)	
Sex				0.394
Male	71 (51.82)	26 (47.27)	45 (54.88)	
Female	66 (48.18)	29 (52.73)	37 (45.12)	
Cochlear hydrops degree				0.068
None	24 (17.52)	12 (21.82)	12 (14.64)	
Mild	45 (32.85)	12 (21.82)	33 (40.24)	
Severe	68 (49.63)	31 (56.36)	37 (45.12)	
Vestibular hydrops degree				< 0.001*
None	16 (11.68)	16 (29.09)	0 (0.00)	
Mild	15 (10.95)	11 (20.00)	4 (4.88)	
Moderate	51 (37.23)	19 (34.55)	32 (39.02)	
Severe	55 (40.14)	9 (16.36)	46 (56.10)	
Perilymphatic enhacement				0.019*
Absent/Cochlea	119 (86.86)	43 (78.18)	76 (92.68)	
Vestibule/Cochleovestibule	18 (13.14)	12 (21.82)	6 (7.32)	
Herniation toward ampulla				0.006*
Absent	88 (64.23)	43 (78.18)	45 (54.88)	
Present	49 (35.77)	12 (21.82)	37 (45.12)	
Symptom onset				0.232
Synchronous	42 (30.66)	15 (27.27)	27 (32.93)	
Auditory symptoms first	58 (42.33)	28 (50.91)	30 (36.59)	
Vestibular symptoms first	37 (27.01)	12 (21.82)	25 (30.48)	
Reason for consultation				0.019*
Unclear	5 (3.67)	4 (7.41)	1 (1.22)	
Falls	1 (0.73)	1 (1.85)	0 (0.00)	
Vertigo	103 (75.74)	37 (68.52)	66 (80.49)	
Fluctuating HL	10 (7.35)	5 (9.26)	5 (6.10)	
Unsteadiness	8 (5.89)	1 (1.85)	7 (8.53)	
BPPV poor evolution	1 (0.73)	0 (0.00)	1 (1.22)	
Progressive HL	8 (5.89)	6 (11.11)	2 (2.44)	
Tumarkin				0.357
Absent	114 (83.21)	48 (87.27)	66 (80.49)	
Present	23 (16.79)	7 (12.73)	16 (19.51)	
Headache				0.778
Absent	103 (75.73)	40 (74.07)	63 (76.83)	
Migraine	24 (17.65)	11 (20.37)	13 (15.85)	
Tension-type	9 (6.62)	3 (5.56)	6 (7.32)	
CV risk factors				0.036*
Absent	105 (77.20)	37 (67.27)	68 (83.95)	
Present	31 (22.80)	18 (32.73)	13 (16.05)	
Spontaneous nystagmus				0.483
Absent	67 (49.26)	29 (53.70)	38 (46.34)	
Present	69 (50.74)	25 (46.30)	44 (53.66)	
Video head impulse test				0.549
Normal	99 (73.88)	41 (77.36)	58 (71.60)	
Abnormal	35 (26.12)	12 (22.64)	23 (28.40)	
Ménière’s disease subgroup				0.044*
Classic	84 (70.94)	29 (61.70)	55 (77.14)	
Delayed	11 (9.40)	8 (17.02)	3 (4.29)	0.027*^
Familiar	2 (1.71)	1 (2.13)	1 (1.43)	
With migraine	11 (9.40)	7 (14.89)	4 (5.71)	
Autoimmune	9 (7.69)	2 (4.26)	7 (10.00)	
Total	137 (100)	55 (40.15)	82 (59.85)	

^*^p < 0.05. Results of Chi-square test with Monte Carlo simulated *p*-values. ^*Post hoc* significant test that explains a significant multicategorical chi-square test. Missing values are not included in this table, since these data have been excluded from the statistical analysis.

**Table 3:** Results of the Mood’s Median test.

Variable	Total		Groups				*p*-value
			Cochleocentric		Non-cochleocentric		
	Median	IQR	Median	IQR	Median	IQR	
Age	56	17	57	19	56	18	0.281
Disease duration	3	7	4	8	3	6	0.250
Endolymphatic ratio	71	50	55	49.50	80.50	38	< 0.001*
Number of vertigo spells^	4	6	4	4	5	5	0.502
Pure tone audiometry	51	31.50	42.50	32.38	56.50	29.72	0.066
Low frequency PTA	55	25	55	22.50	55	30	0.280
High frequency (4-8 Hz) PTA	60	21.70	55	29.15	60	19.50	0.313

^*^p < 0.05; IQR, Interquartile range. ^Number of vertigo spells in the 6 months previous to evaluation.

### MRI findings

**Figure 3:**
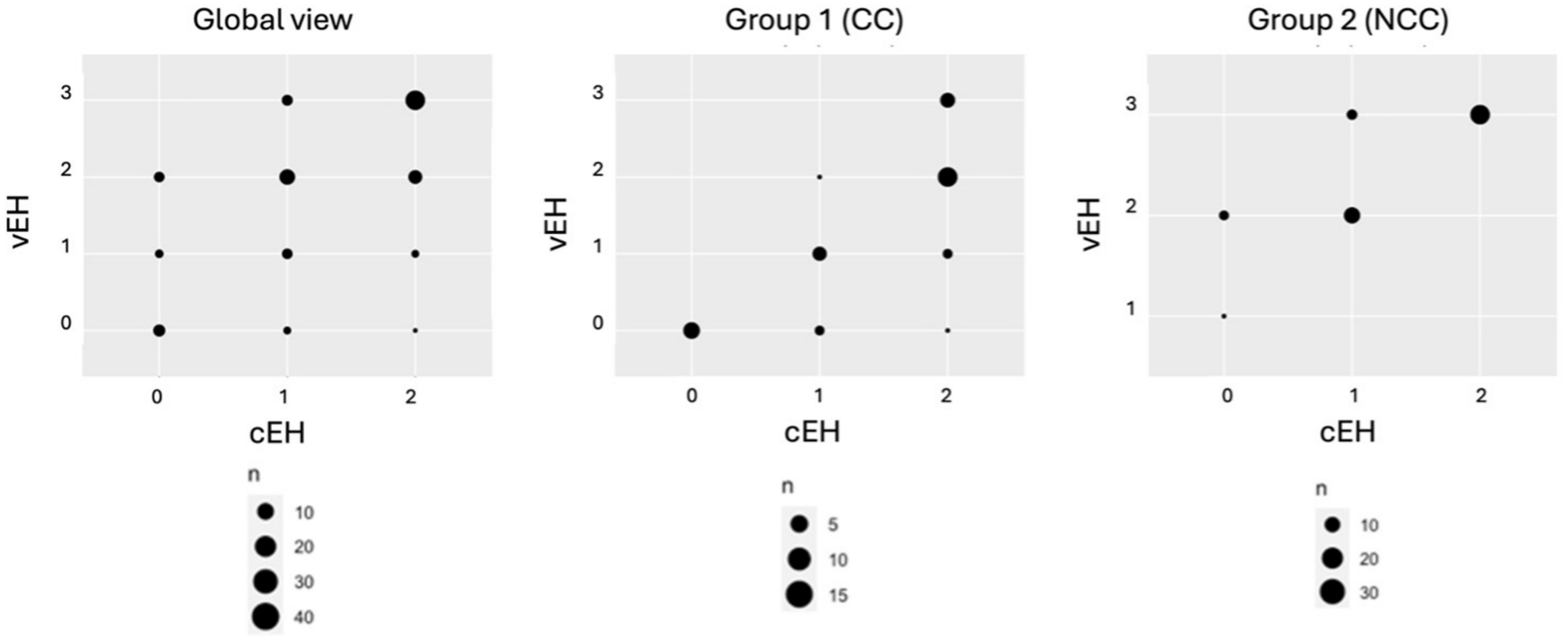
Inter- and intra-group correlation between vestibular (vEH) and cochlear (cEH) hydrops in the affected ear. The patients were categorized by the degree of EH in the vestibule (0–3) and cochlea (0–2). Group 1 is the cochleocentric (CC) group and Group 2 is the non-cochleocentric (NCC) group.

The degree of vEH on the affected side was more severe in the patients belonging to the NCC group (*p* < 0.001). Although cEH on the affected side did not show significant differences between the groups (*p* = 0.068) (Table 2), it almost reached significance and was moderately correlated with the degree of vEH (*Kendall’s Tau-b* = 0.59, *p* < 0.001). This correlation increased when the patients were separated into the CC group (*Kendall’s Tau-b* = 0.78, *p* < 0.001) and the NCC group (*Kendall’s Tau-b* = 0.80, *p* < 0.001) (Figure 3).

**Figure 4:**
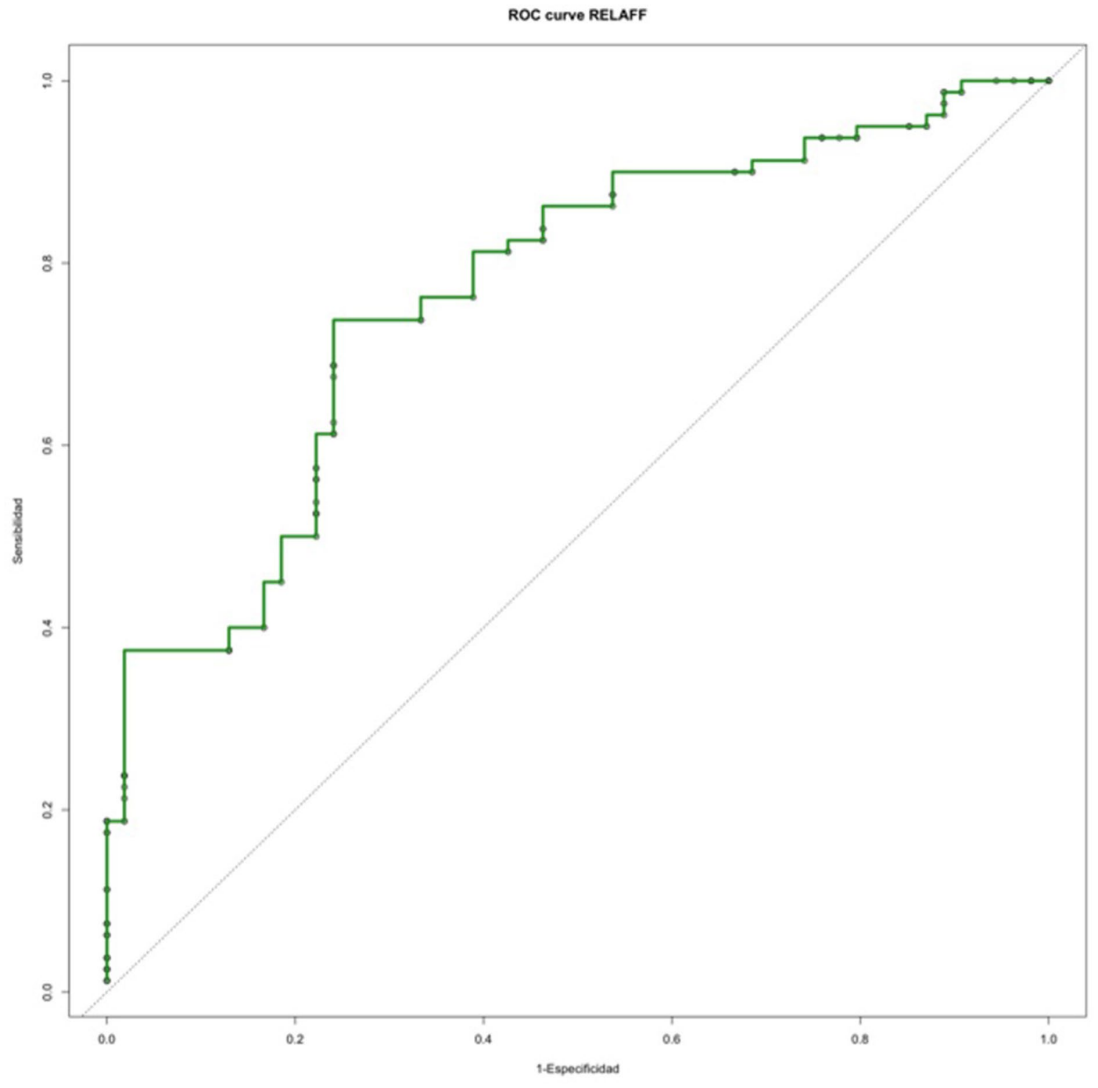
ROC curve analyzing vELR.

On the affected side, the mean vELR was 71% ± 50%, with 55% ± 49.50% in the CC group and 80.50% ± 38% in the NCC group. A higher vELR value on the affected side was associated with the NCC group (*p* < 0.0001) (Table 3). The classification power of this potential biomarker to distinguish between the two groups was also assessed using a ROC curve (Figure 4). The optimal cut-off point, estimated by the Youden Index (J), was 0.67 (sensitivity = 0.69; specificity = 0.76).

Finally, the presence of vestibular herniation into the ampulla was more likely to be found in the NCC group (*p* = 0.006), while the presence of vestibular perilymphatic enhancement was statistically more prevalent in the CC group (*p* = 0.019) (Table 2).

### Past medical history and clinical features

The patients with cardiovascular risk tended to belong to the CC group (*p* = 0.036). The other associated comorbidities studied did not show relevant results (headache: *p* = 0.778; arterial hypertension: *p* = 0.701; diabetes mellitus: *p* = 1; hypercholesterolemia: *p* = 0.611) (Table 2).

According to the reason for the first consultation, the patients in the CC group reported more symptoms related to hearing impairment (fluctuating hearing loss and progressive hearing loss), while the patients in the NCC group consulted more frequently for vestibular impairment (falls, unsteadiness, vertigo, and benign paroxysmal positional vertigo with poor evolution) (*p* = 0.038) (Table 2).

The results also showed statistically significant differences between the different subgroups of Ménière’s disease (*p* = 0.04). The *post hoc* analysis revealed that this difference was found in the delayed form, which was associated with the CC group (*p* = 0.027) (Table 2).

The rest of the variables studied did not show statistically significant results (disease duration: *p* = 0.250; number of vertigo spells in the 6 months prior to evaluation: *p* = 0.502; spontaneous nystagmus: *p* = 0.483; symptom onset: *p* = 0.232; Tumarkin’s otolithic crisis: *p* = 0.357; and days since the last vertigo spell: *p* = 0.737) (Tables 2–4).

**Table 4:** Results of the Mann–Whitney U test.

Variable	Total		Groups				*P-*value
			Cochleocentric		Non-cochleocentric		
	Mean	SD	Mean	SD	Mean	SD	
Days since last crisis	48	88	54	98	45	81	0.738
cVEMP asymmetry	36.31	28.29	33.83	25.52	37.83	29.76	0.621
oVEMP asymmetry	36.15	26.99	32.78	25.58	38.20	27.62	0.331

SD, Standard deviation.

### Hearing loss and vestibular function

The PTA could not differentiate between the CC and NCC groups (*p =* 0.066), nor did the audiometric levels for the low (bfaff) and high (AFF4a8) frequencies show statistically significant differences (*p* = 0.280 and *p* = 0.313, respectively) (Table 3).

None of the vestibular function tests (VEMP and vHIT) showed statistically significant differences between the groups, as seen in Tables 2, 4.

## Discussion

In this study, we found that the degree of EH was more severe in the vestibule than in the cochlea in almost 60% of the cases and that there was a clinical correlation with the symptoms that initiated the disorder. However, in the follow-up, there were no significant differences in either the auditory or vestibular function tests. In a pioneering study, previous authors have shown (using different sequences and administering contrast media via the intratympanic route) that in patients with unilateral definite MD, vEH occurs very early in the disease and its progression subsequently heralds involvement in the cochlea (22). Our work was prompted by the finding that contradicts the knowledge from experimental and clinical otopathology. There are two major arguments that support the cochleocentric progression of hydrops. The first argument is the results obtained from surgically induced hydrops by damaging the endolymphatic sac in the guinea pigs (23) and the second is the distribution of the histological lesions secondary to hydrops or rupture of labyrinthine membranes (24). In both cases, the order of progression and frequency are higher in the apex of the cochlea than in its base, followed by the saccule, utricle, and, finally, the ampullae. Contrary to this, otopathological records from other experimental hydrops models, such as genetic (25) or autoimmune (26) models, as well as cases of secondary EH (27), show a different severity of hydrops, where cEH is not always as severe as vEH (28).

With the advent of MRI techniques to visualize EH, the number of patients with documented EH has increased significantly. Using this methodology, it has been shown by several research groups that the total volume of endolymph and its relative value (to total fluid space) are significantly higher in patients with unilateral MD compared to controls. Interestingly, as recently shown in a very detailed study, the relative amount of endolymph is slightly higher in the vestibule than in the cochlea: 36.8% ± 21.4 and 22.3% ± 12.4%, respectively (29).

Based on these recent arguments, our work yielded plausible results. Nonetheless, we must consider the possibility that extreme differences in the degree of EH between the cochlea and vestibule may be due to intrinsic damage to the inner ear (membrane collapse) or an assessment artifact. The collapse of the endolymphatic space is evident in MRI hydrops assessment sequences, where the complete absence of endolymph is observed, leaving only the contrast visible in the evaluated region (cochlea or vestibule) (30). However, some authors have argued that the adequate visualization of findings, such as this collapse, requires enhanced ultrahigh-resolution hydrops MRI (31). Notably, this phenomenon can also occur in specific parts of the membranous labyrinth, such as the saccule (32). In early papers from 2016, the group of Inui et al. described four patterns of endolymphatic space visualization, either in the vestibule or cochlea, but in patients without Ménière’s disease. One of their variants included the absence of endolymph visualization in both the cochlea and the vestibule, with the former being the most frequent (33). In our study, we did not find any case of possible membrane collapse. However, in 13/137 patients, we observed a very significant difference in the degree of EH as qualitatively assessed in the cochlea and vestibule: 4/55 (7.2%) patients in the CC group were diagnosed with cEH grade 2 and vEH grades 0 or 1, while 9/82 (10.9%) patients in the NCC group had cEH grade 1 and vEH grade 3. In these patients, we can speculate that some recovery of the endolymphatic space occurred after a rupture, as seen in vertigo attacks (34) or post-operative perilymphatic fistula repair (30). However, in our patients, we did not observe any significant differences either in the number of vertigo crises in the last 6 months or in the days since the last vertigo crisis prior to the MRI evaluation (Table 4).

We considered three different technical limiting factors that could lead to improper EH visualization, some of which are more specific to cEH. One factor is the higher spatial resolution required to visualize the cochlear ultrastructure properly. It is important to note that 3D-FLAIR is the most widely used sequence for hydrops imaging. However, some *in vitro* experiments have reported that T2-contrast enhancement techniques should be used with caution in 3D-FLAIR for diagnosing endolymphatic hydrops (34) and that this sequence is more sensitive to small Gd concentrations in endolymph and to the inversion time employed (35). To overcome these issues, we choose to use the 3D-REAL-IR sequence for the hydrops imaging as it has shown to be a superior sequence in assessing cochlear EH (13), a finding later replicated in other studies (36). Motion artifacts are common in any MRI study, and reducing the assessment time by focusing on the most relevant sequences allowed us to exclude only one case before inclusion. In addition, no other artifacts have been described in the 3D-REAL-IR sequence that could reduce the visualization of endolymph in the cochlear duct. A second possible limitation of our 3D-REAL-IR sequence is the slice thickness (0.8 mm), compared to the cisternography sequence (0.5 mm). Thinner slices would be ideal, and future research may employ a 0.5 mm slice thickness using image acquisition acceleration techniques and high-density phased-array head coils.

Another potential limitation of our study is the use of qualitative scales for classifying the cochleocentric and non-cochleocentric groups, utilizing a 3-grade scale for cEH and a 4-grade scale for vEH. In future research, volumetric quantification with the calculation of vestibular and cochlear endolymphatic ratios can undoubtedly allow for a more accurate classification of these groups, especially in cases where qualitative grades overlap. In addition, Kirsch’s working group recently described the role of MRI-based EH visualization techniques in diagnosing vestibular migraine, highlighting key differences in EH distribution between these patients and those with Ménière’s disease. Specifically, in patients with vestibular migraine, the intralabyrinthine distribution was predominantly observed in the vestibule, the hydrops tended to be bilateral, and the degree of EH often correlated with the frequency and duration of episodes (37). In our sample, a total of 137 patients were included, of whom only 24 reported migraine-like headaches. These patients were distributed evenly between the CC (11 patients) and NCC (13 patients) groups. While the association of migraine-like headaches with MD in certain patients could potentially introduce classification bias, given the recent findings from Kirsch et al., the small sample size of these cases in our study, along with the similar distribution in both CC and NCC groups among the patients who presented with migraine-type headaches, and the lack of statistical significance suggest that this factor is not a limiting one in our study. However, we recommend conducting a future study that directly compares patients diagnosed with MD and those with confirmed vestibular migraine. Such a comparative study could provide a clearer characterization of EH patterns observed on MRI in these two distinct groups, allowing for a more precise assessment of potential diagnostic markers specific to each condition.

In Table 5, we present a summary of the clinical findings. When analyzing the different phenotypes of MD, the “delayed” phenotype was associated with the cochleocentric group likely because these patients experienced the first damage as hearing loss (3/11) or sudden sensorineural hearing loss (5/11) at least 15 years before the vestibular symptoms occurred. In 2/11 cases, hearing loss fluctuated for a long period without vertigo. This is also consistent with the main initiating events in the patients’ clinical history and their main concern when first visiting after the initial vertigo episodes: all of them were more worried about hearing loss than vertigo. In addition, cardiovascular risk factors were more prevalent, which was expected as sensitivity to ischemia varies across different tissues in the inner ear, with the standing hair cells in the organ of Corti and the stria vascularis being the most vulnerable in the cases of arterial or venous damage (38). In this group, we consider EH as a phenomenon governed by the function of the utriculo-endolymphatic valve of Bast (9), which is related to the morphology of the vestibular aqueduct’s opening to the endolymphatic sac. In case this makes an open (>140°) angle (39), then the role of Bast’s valve becomes highly relevant in controlling the reflux of endolymph. However, initiating damage may make the cochlear region more susceptible to injury. This localized damage could result from otoconia freely floating in the saccule, which may eventually obstruct the opening of Hensen’s duct (ductus reuniens), the structure that connects the cochlea and saccule (8, 40). Perilymphatic enhancement can be considered an indicator of increased activity at this site.

**Table 5:** Summary of the findings.

	Cochleocentric	Non-cochleocentric
Degree of vestibular hydrops	Low	High
Vestibular Endolymphatic ratio	< 67%	> 67%
Perilymphatic enhacement	Yes	No
Vestibular herniation to the ampulla	No	Yes
Reason for the first consultation	Hearing impairment	Vestibular impairment
Cardiovascular risk factors	Yes	No
Subgroup of Ménière’s disease	Delayed	

In the non-cochleocentric group, the initial symptoms were mainly vestibular, and during the MRI assessment, all vestibular results of the NCC group were worse than those of the CC group. In this group, endolymphatic herniation into the semicircular canals was more frequently observed compared to the cochleocentric group. According to otopathological records, herniation primarily results from the progression of the endolymphatic terminal of the cochlea, known as the “vestibular cecum of the cochlea.” We believe that this may be another reason for the lower degree of cEH as this structure has thin, flat, and anti-elastic walls, similar to those of the cochlear duct and saccule. According to authors, this indicates a high proclivity for stress and hydropic distension (41). We believe this acts as a mechanism to reduce distension in the basal cochlear duct, thereby reducing the level of EH. However, with actual MRI technology, it is not possible to identify which structure occupies the vestibule in cases of severe hydrops (32).

It is tempting to speculate that inflammatory processes could be the primary cause in the NCC group, initiating malfunction at the vestibule. We believe that the structural evaluation of the temporal bone and the distribution of EH provides valuable insights into certain characteristics of the disease and offers a partial answer to the crucial question about the role of EH in MD. By properly selecting the phenotype (42) and combining the structural study with the analysis of various inflammatory markers that are associated with the onset (6), chronicity, and recurrence (5), we can gain a better understanding of this disorder.

## Data Availability

The raw data supporting the conclusions of this article will be made available by the authors, without undue reservation.
